# A Flexible Triboelectric-Based Sensor for Seismocardiography Monitoring

**DOI:** 10.3390/bios16050260

**Published:** 2026-05-01

**Authors:** Changke Wang, Yingjie He, Haojie Peng, Haijun Luo, Xue Wang

**Affiliations:** College of Physics and Optoelectronic Engineering, Chongqing Normal University, Chongqing 401331, China

**Keywords:** flexible pressure sensor, triboelectric nanogenerator, SCG

## Abstract

Seismocardiography (SCG) is a promising noninvasive modality for cardiovascular monitoring. By capturing subtle chest wall vibrations induced by the mechanical pumping activity of the heart at the body surface, SCG is of considerable value for blood pressure-related cardiovascular risk assessment and cardiac function monitoring. However, continuous SCG monitoring in daily life settings still relies predominantly on rigid accelerometers, and reports on flexible acquisition systems remain scarce. This is mainly because SCG signals are characterized by low frequency, low amplitude, and high sensitivity to the sensor-skin interface, requiring the sensor to achieve stable, high-fidelity acquisition of weak chest wall mechanical vibrations while maintaining conformal contact and wearing comfort. To address this challenge, this study proposes a flexible pressure sensor based on the triboelectric effect. The sensor adopts a single-electrode contact-separation structure and is composed of a polymer material capable of achieving a high negative charge density and a nickel foil electrode. The sensor exhibits a sensitivity of 3.76 V/N within a small force range of 0–200 mN, shows good frequency response over the 0.5–25 Hz band, and maintains stable output after approximately 5300 cycles. The sensor was attached to the lower-middle segment of the sternum to capture weak vibration signals generated by cardiac mechanical activity and transmitted through the chest wall, thereby enabling continuous SCG monitoring. This study presents a feasible approach for flexible SCG acquisition in daily life scenarios and provides experimental evidence supporting the application of flexible sensors in home-based health monitoring.

## 1. Introduction

Cardiovascular disease remains a leading cause of mortality worldwide and a major contributor to global disease burden. Therefore, the development of continuous monitoring technologies capable of reflecting cardiac functional status is of considerable importance. Continuous monitoring of cardiac mechanical activity can provide dynamic information on cardiac contraction and relaxation and facilitate the early identification of potential abnormalities in cardiovascular function. As a noninvasive technique that records subtle vibrations on the chest wall induced by cardiac mechanical activity, seismocardiography (SCG) can characterize key mechanical events during the cardiac cycle [1,2], including aortic valve opening and closure (AO/AC) [3,4], as well as vibratory features associated with isovolumetric contraction and diastolic filling [5,6,7]. Based on the temporal information of these events, SCG can be further used to estimate cardiovascular hemodynamic indices such as the pre-ejection period (PEP) and left ventricular ejection time (LVET), thereby providing a potential basis for assessing myocardial contractility, afterload, stroke volume, and arterial compliance [8,9,10]. Because SCG signals are modulated by the cardiac-arterial coupling mechanism, their key features and temporal parameters are responsive to blood pressure-related hemodynamic changes. Consequently, SCG has shown considerable potential in the monitoring and evaluation of various cardiovascular diseases, including heart failure, cardiac dysfunction, and arrhythmia [11,12,13,14].

However, existing SCG acquisition systems still rely predominantly on rigid sensors such as MEMS accelerometers [15,16,17], thereby imposing multiple limitations in wearable continuous-monitoring scenarios. First, rigid devices are not mechanically compatible with the soft and continuously moving chest wall biointerface. Under the influence of respiration, postural changes, skin displacement, perspiration, or friction from clothing, micro-displacements can readily occur at the sensor-skin interface, thereby leading to signal amplitude drift, waveform distortion, and reduced consistency across repeated measurements [18]. Second, accelerometers inherently output the projection of acceleration along the sensitive axis, whereas SCG signals exhibit pronounced spatial heterogeneity and directional dependence. Therefore, slight positional shifts, changes in attachment angle, or errors in mounting orientation may attenuate the amplitudes of key mechanical events [19]. In addition, rigid devices generally struggle to simultaneously maintain conformal contact, wearing comfort, and interfacial stability during long-term wear, which further limits their application in home-based health monitoring and daily life scenarios. Therefore, developing a novel flexible SCG sensing strategy that combines wearing comfort, interfacial stability, and high-fidelity signal acquisition has become a key issue in advancing SCG toward long-term and routine daily use.

To address the above limitations, this study proposes a flexible pressure sensor based on a triboelectric nanogenerator (TENG). Furthermore, by integrating the sensor with a signal-conditioning circuit and a host computer, we constructed a Miniaturized Triboelectric Seismocardiography System (MTSS) for continuous SCG acquisition. The sensor adopts a single-electrode contact-separation architecture, in which highly electronegative PTFE is selected as the triboelectric layer to achieve a high surface charge density, while a wear-resistant, high-strength nickel foil is used as the counter triboelectric layer and simultaneously serves as the electrode, thereby enhancing system integration and the stability of charge collection [20,21,22,23,24,25,26,27,28]. Performance evaluation showed that the sensor exhibited a sensitivity of 3.76 V/N over the 0–200 mN range, a frequency response spanning 0.5–25 Hz, and stable output after approximately 5300 cycles. The sensor was then integrated into a wearable strap and fixed to the lower-middle segment of the sternum, enabling SCG signal acquisition across different individuals and under various daily life conditions. Experimental results demonstrated that the MTSS was capable of acquiring SCG waveforms with clear morphology and identifying key features related to AO and the isovolumetric phases. Further preliminary analysis showed that the three-class classification models for SBP and DBP based on SCG feature parameters achieved accuracies of 99% and 97%, respectively. These findings indicate that a TENG-based flexible sensor, combined with a system-level integration strategy, holds promise as a new approach for comfortable, long-term SCG monitoring and blood pressure-related risk screening. Future work will further improve model generalizability and evaluate its application potential in real-world home settings through algorithm optimization and validation using larger-scale datasets.

## 2. Materials and Methods

### 2.1. Working Principle of the Miniaturized Triboelectric Seismocardiography System

**Figure 1:**
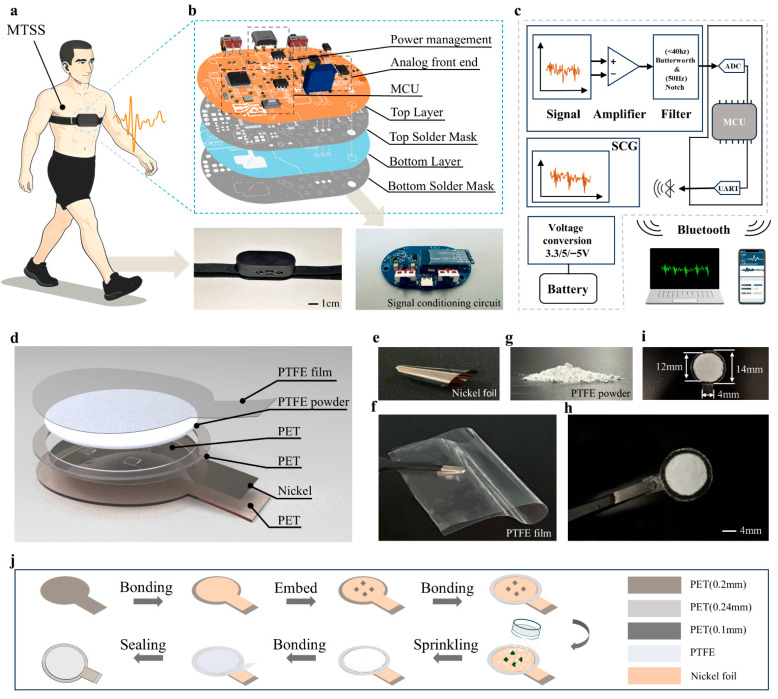
Flexible triboelectric sensor and system for continuous SCG monitoring. (**a**) Schematic illustration of the MTSS for SCG monitoring in daily life settings. (**b**) Signal-conditioning circuit designed for seismocardiographic signals. (**c**) Complete system architecture and signal transmission pathway. (**d**) Structural composition of the flexible pressure sensor. (**e**) Nickel foil used as the triboelectric layer and electrode material. (**f**) PTFE film used as the contact layer. (**g**) PTFE powder used as the triboelectric material. (**h**) Photograph of the assembled flexible pressure sensor. (**i**) Dimensions of the flexible pressure sensor. (**j**) Fabrication process of the flexible pressure sensor.

As shown in Figure 1a, the MTSS can be used for continuous monitoring of SCG signals in daily life settings. The system integrates a flexible sensor, a signal-conditioning circuit specifically designed for SCG signals, and a host computer for SCG waveform display. As shown in Figure S1a, to ensure that the flexible sensor remained stably positioned at the lower-middle segment of the sternum during measurement, a Velcro strap was used for fixation in this study. Given that the sensor adopts a single-electrode structure, the human body is required to serve as a reference ground to form a stable electrical circuit. Therefore, conductive fibers were sewn onto the inner side of the Velcro strap so that they could contact the skin and provide a body reference ground (Figure S1b).

As shown in Figure 1b, the signal-conditioning circuit mainly consists of three parts: a power management module, an analog signal processing module (analog front end), and an MCU subsystem. For power management, the LGS5500EP (Legend-Si, Shenzhen, China) was used for charging management of a 3.7 V lithium battery. The battery output was then regulated to a fixed 5 V to provide a stable power supply for the entire system. Subsequently, the TP7660H (TOPPOWER, Nanjing, China) and ME6206A33M3G (MICRONE, Nanjing, China) were used to convert the 5 V supply into −5 V and 3.3 V, respectively, thereby providing the required dual supply voltage and logic supply voltage for the subsequent analog front-end operational amplifiers and MCU subsystem. In the analog signal processing chain, the current signal generated by the sensor was first fed into a transimpedance amplifier circuit constructed using the LMC6482IM (Texas Instruments (TI) Inc., Dallas, TX, USA) to achieve current-to-voltage conversion. A 10 MΩ resistor was used as the transimpedance feedback resistor, and a small capacitor was connected in parallel across it to suppress high-frequency noise, improve stability, and prevent oscillation; in this study, a 20 pF capacitor was selected. The signal then entered a third-order Butterworth low-pass filter (Figure S2a). Considering that the main frequency components of the seismocardiographic signal are concentrated within 1–20 Hz, the low-pass cutoff frequency was set to 33.9 Hz (Figure S2b) in this study to suppress high-frequency noise while preserving effective signal details as much as possible. This cutoff frequency was chosen to cover the main energy distribution of SCG (1–20 Hz) while retaining a certain margin. After filtering, the signal was further amplified by an amplifier circuit based on the GS8552-SR (Gainsil, Shanghai, China). Because the single-electrode sensor structure is more susceptible to power-line interference, a 50 Hz notch filter was placed immediately after the amplification stage to suppress mains-frequency noise. Finally, level shifting was achieved through a series capacitor and a voltage-divider network, which also formed a high-pass characteristic of approximately 0.8 Hz to suppress baseline drift and ultra-low-frequency artifacts, such as slow postural changes and respiration-related low-frequency components, thereby enhancing the observability of cardiac vibration-related components. The processed signal was then sent to the ADC input of the MCU and sampled at 1000 Hz. The STM32F103C8T6 (STMicroelectronics, Geneva, Switzerland) was selected as the main control chip. Its built-in 12-bit ADC has a minimum voltage resolution of 0.732 mV, which is sufficient to meet the acquisition requirements of this study.

The sampled data were transmitted through UART to a Bluetooth module and received on the host computer in the form of a virtual COM port. On the host compute, Python (v3.12) was used to read the serial data and forward them to a local WebSocket service. On the web page side, a WebSocket connection was established using HTML/JavaScript to enable real-time data reception and visualization, and the schematic structure is shown in Figure 1c. The coordinated design of the above hardware and data transmission chain helps enable long-term, stable, and real-time monitoring of SCG signals.

### 2.2. Sensor Design and Material Selection

Seismocardiography (SCG) refers to the weak vibratory waveform formed by the transmission of mechanical vibrations generated by the heart during each contraction and relaxation through the chest wall. To meet the requirements for wearable acquisition of subtle chest wall vibrations, this study developed a single-electrode contact-separation triboelectric sensor for SCG monitoring. As shown in Figure 1d, the device has a layered structure. From the skin side outward, it consists of a skin-contact layer, a triboelectric functional layer, a gap-support layer (introduced to create a controllable contact/separation gap), and a flexible substrate layer. A 200 μm polyethylene terephthalate (PET) film (Figure S3a) was selected as the device substrate to balance flexibility and structural stability. The conductive layer was a 12 μm PET-based composite nickel foil (Figure 1e). Its PET side shows good interfacial compatibility with the PET substrate, facilitating attachment and fixation, while the nickel side serves as the electrode and provides excellent corrosion resistance, wear resistance, and oxidation resistance.

The materials were selected by comprehensively considering the triboelectric series and charge-storage characteristics. The triboelectric functional layer consisted of a combination of a 30 μm transparent polytetrafluoroethylene (PTFE) film (Figure 1f) and PTFE powder with an average particle size of 3 μm (Figure 1g). PTFE exhibits a typical negative triboelectric polarity and strong charge-retention capability, which is beneficial for obtaining stable and repeatable output signals. In addition, the PTFE film possesses hydrophobicity and chemical corrosion resistance. When used as the skin-side contact layer, it helps improve durability and wearing comfort in wearable applications. These properties enable the PTFE layer, when paired with nickel as the triboelectric counterpart, to provide stable mechanical performance and a long service life.

The supporting spacer layer was formed using PET double-sided adhesive tape (Figure S3b) to ensure material compatibility, and 100 μm PET square pads (Figure S3c) were introduced as local support points. These pads were positioned in the four quadrants of the circular effective area to ensure sufficient separation of the triboelectric interfaces during loading and unloading, thereby reducing signal drift caused by adhesion effects and improving cyclic consistency and repeatability. The fabricated sensor is shown in Figure 1h.

### 2.3. Sensor Fabrication Process

As shown in Figure 1i, the 200 μm PET substrate film and PTFE film were first cut into specific shapes, with a circular region of 14 mm in diameter and a lead-out tab measuring 5 mm in length and 4 mm in width. The PET-based composite nickel foil was cut into the same overall shape, with a circular region of 12 mm in diameter and a lead-out tab measuring 4 mm in length and 3 mm in width. Subsequently, PET double-sided adhesive tape was attached to release paper and cut into a ring structure with an outer diameter of 14 mm and an inner diameter of 12 mm. In addition, a 100 μm PET film was cut into four square pads, each with an area of 1 mm^2^. All cut materials were cleaned with ethanol and allowed to dry naturally.

After drying, the nickel surface of the nickel foil was lightly polished with sandpaper to modify the surface morphology. Subsequently, a room-temperature-curing silicone adhesive was used to bond the PET side of the nickel foil to the precut PET substrate. After the adhesive layer had fully cured, the four PET pads were uniformly attached to the four quadrant positions of the circular effective area on the nickel surface. Next, the PET double-sided adhesive ring was aligned with and attached along the outer edge of the circular region of the nickel foil to form a stable limiting and supporting structure. PTFE powder with an average particle size of 3 μm was then uniformly distributed over the effective area using a mesh screen. Its distribution and microstructure under SEM are shown in Figure S4a,b. Finally, the precut PTFE film was overlaid and attached to complete the device assembly (Figure 1j).

## 3. Results and Discussion

### 3.1. Working Principle of the Sensor

**Figure 2:**
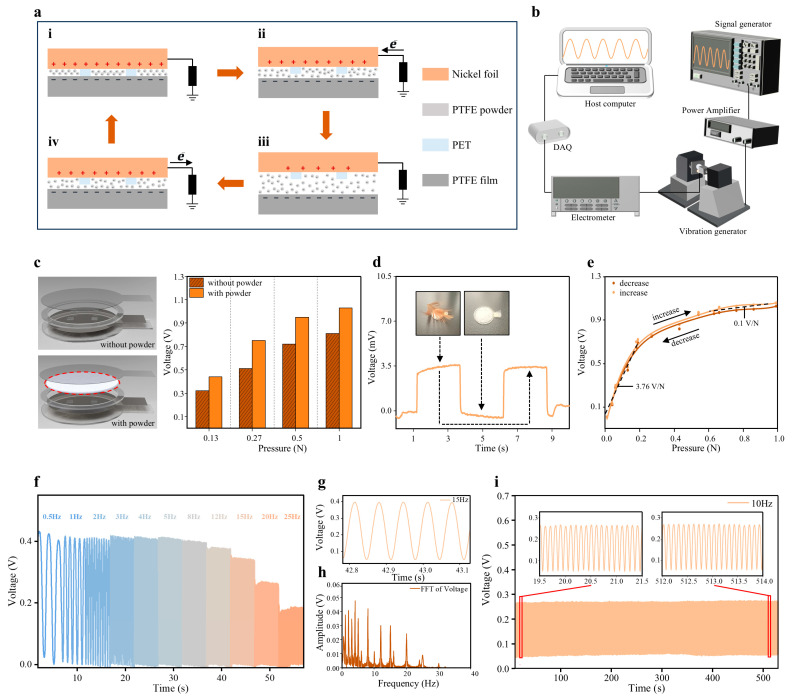
Working principle and performance evaluation of the sensor. (**a**) Working principle of the single-electrode contact-separation triboelectric sensor: (**i**) original state; (**ii**) The gradual increase in the distance between the two contact surfaces; (**iii**) The two contact surfaces have achieved the maximum gap; (**iv**) The gap distance between the two contact surfaces gradually returned to its original state. (**b**) Performance testing platform. (**c**) Output amplitudes of the sensors with and without PTFE powder under different applied forces (0.13, 0.27, 0.5, and 1 N). (**d**) Response of the flexible pressure sensor to slight force variations. (**e**) Response of the flexible pressure sensor to forces of different magnitudes applied in loading and unloading sequences. (**f**) Frequency response of the flexible pressure sensor to low-frequency signals (0.5–25 Hz). (**g**) Undistorted waveform of the flexible pressure sensor at 15 Hz. (**h**) Frequency spectrum of the output signal of the flexible pressure sensor. (**i**) Output voltage of the flexible pressure sensor over approximately 5300 cycles, with no significant performance degradation.

The output of the single-electrode triboelectric nanogenerator sensor originates from the coupled effects of contact electrification and electrostatic induction. As shown in Figure 2a, in the initial state, the nickel electrode and the PTFE powder/PTFE film are brought into full contact under an external mechanical force. Owing to the difference in electron affinity between the two materials, charge transfer occurs at the interface after contact: PTFE has a stronger tendency to gain electrons and therefore accumulates negative charges on the contact surface, whereas nickel correspondingly loses electrons and carries an equal amount of positive charge. It should be noted that, as a polymer insulating material, PTFE exhibits slow surface charge decay, allowing the negative charges to remain stably on the surface for a relatively long period, ranging from hours to even days. When the two layers are in complete contact, and the triboelectric charges are mainly distributed on the same interfacial plane, the system externally exhibits a potential difference close to zero [Figure 2a(i)].

When an external mechanical motion creates a gap between the two triboelectric layers—for example, when subtle skin vibrations on the lower-middle segment of the sternum induced by cardiac mechanical activity drive the separation of the triboelectric layers—a potential difference is established between the two electrodes because of electrostatic induction. If the external circuit is open, this potential difference can be defined as the open-circuit voltage, V_oc_. As the separation distance increases, V_oc_ gradually rises and approaches saturation when the gap reaches its maximum. Once the electrode is connected to the external circuit, the established potential difference drives directional electron transfer through the external circuit: electrons flow from the reference electrode (with the human body serving as the reference ground) to the main nickel electrode, thereby generating a current in the external circuit [Figure 2a(ii)]. When the PTFE layer continues to move away to a sufficient distance, the system reaches a new electrostatic equilibrium, and part of the induced charges on the reference electrode is transferred to the main electrode [Figure 2a(iii)].

Subsequently, under the action of an external force, the PTFE layer moves back toward the nickel electrode until contact is re-established. As the separation gap decreases, the direction of the potential difference reverses, and the potential of the main electrode becomes lower than that of the reference electrode. Electrons then flow back from the main electrode to the reference electrode [Figure 2a(iv)], producing a reverse current in the external circuit. Finally, when the two triboelectric layers return to full contact, the charges redistribute, and the system returns to its initial state, thus completing one full contact-separation cycle.

### 3.2. Performance Evaluation of the Flexible Pressure Sensor

To quantitatively evaluate the dynamic responsiveness of the flexible pressure sensor, we constructed the test system shown in Figure 2b. This system consisted of a signal generator (DSG815, Keysight Technologies Inc., Santa Rosa, CA, USA), a power amplifier (LM1875, Texas Instruments Inc., Dallas, TX, USA), a shaker (SA-JZ002, Wuxi Shiao Technology Co., Ltd., Wuxi, China), an electrometer (Keithley 6514, Tektronix Inc., Beaverton, OR, USA), a data acquisition card (NI USB 6008, National Instruments Inc., Austin, TX, USA), and a host computer. Before testing, the sensor under test was fixed onto the shaker. The sensor electrode was connected to the electrometer through a wire, with the test bench serving as the reference ground. During the experiment, the output signal from the signal generator was amplified by the power amplifier to drive the shaker. The output signal from the electrometer was collected by the data acquisition card and uploaded to the host computer for real-time waveform display and data storage.

First, to evaluate the effect of PTFE powder on sensor performance, we tested two different sensor designs, namely, one without PTFE powder and one with PTFE powder. Using the constructed test system, rectangular-wave inputs with different amplitudes were applied to the shaker to exert different force levels on the two sensors, and the output voltage generated at each force level was recorded. As shown in Figure 2c, under the four applied force levels (0.13 N, 0.27 N, 0.5 N, and 1 N), the sensor with PTFE powder produced markedly higher output voltages than the sensor without PTFE powder. These results indicate that the introduction of an appropriate amount of PTFE powder can increase the effective contact area of the triboelectric interface and enhance the amount of charge generated by contact electrification, thereby significantly improving sensor sensitivity.

Subsequently, we further investigated the effect of PTFE powder loading on device performance. To this end, flexible pressure sensors containing 20 mg, 40 mg, and 60 mg of PTFE powder were fabricated, respectively. As shown in Figure S5, the preliminary results indicate that the device incorporating 40 mg of PTFE exhibits higher sensitivity than the other two groups. This phenomenon may be closely related to the efficiency of charge generation and transfer at the triboelectric interface. Specifically, when the PTFE powder content is too low, the effective interface available for triboelectrification is insufficient, making it difficult to generate adequate charges during the contact–separation process. Conversely, when the PTFE powder content is excessive, the surplus powder may decrease the contact gap and weaken the actual interfacial contact, thereby reducing the efficiency of charge transfer and accumulation, ultimately leading to a decrease in output voltage.

Accordingly, the flexible pressure sensor containing 40 mg of PTFE powder was selected for electrical characterization. The short-circuit current increased nearly linearly with increasing applied pressure (Figure S6). To further evaluate the output performance, the sensor was connected to external loads with different resistances and tested under a fixed force amplitude and frequency. The peak output voltage was recorded, and the corresponding output power was calculated from the measured voltage (Figure S7). As shown in Figure S7, the maximum output power reached approximately 2.5 nW at a load resistance close to 10^8^ Ω.

Given that SCG signals essentially originate from weak mechanical vibrations generated by the heart, we then placed the flexible pressure sensor flat on the test bench, connected the sensor electrode layer to the electrometer, and used the bench as the reference ground. A feather was picked up with tweezers and placed onto the sensor repeatedly while the voltage variation was recorded. As shown in Figure 2d, when the feather was placed on the flexible pressure sensor, the output voltage was approximately 3.5 mV. This result further demonstrates that the flexible pressure sensor has a clear and distinguishable response to slight force variations. Meanwhile, the response and recovery times of the sensor were systematically evaluated (Figure S8). The results show that the response time reaches 0.02 s, while the recovery time is 0.06 s, indicating favorable dynamic response characteristics, which enable accurate detection of seismocardiogram signals.

One SCG cycle usually corresponds to a complete cardiac cycle, that is, the process from one cardiac contraction to the next. To evaluate the sensor response, forces of different magnitudes were applied in loading and unloading sequences, respectively, and the corresponding output voltages were recorded, yielding the two fitted curves shown in Figure 2e. The fitting results showed that the sensitivity was 3.76 V/N in the range of 0–0.2 N, whereas it decreased to 0.10 V/N in the range of 0.6–0.8 N, indicating that the flexible pressure sensor was more sensitive to slight force changes. This decrease in sensitivity with increasing force mainly arises because the contact interface gradually approaches full contact, causing the increments in effective contact area and accumulated triboelectric charge to approach saturation. Meanwhile, the structure becomes compressed and stiffened under higher loads, so that the deformation and electrical response induced by each incremental force decrease, leading to a reduced slope in the output-force relationship.

Next, based on the main frequency range of SCG signals, we tested the frequency-response performance of the flexible pressure sensor. During the test, the signal generator output sinusoidal waves with a fixed amplitude and varying frequencies, with the frequency switched every 5 s. The results are shown in Figure 2f. Over the frequency range from 0.5 to 25 Hz (Figure 2h), the recorded waveforms showed no distortion, as shown in Figure 2g, verifying that the flexible pressure sensor can faithfully reproduce the waveform of SCG signals.

Considering that the application scenarios of the MTSS typically involve prolonged wear, we also conducted a stability test of the sensor. The signal generator output a sinusoidal wave at 10 Hz, and after approximately 5300 cycles, the magnified waveforms at the beginning and the end of the test showed no distortion, as shown in Figure 2i. These results indicate that the flexible pressure sensor is capable of stable long-term operation while maintaining good mechanical durability.

### 3.3. Comparison with SCG Signals Acquired by a MEMS Sensor

**Figure 3:**
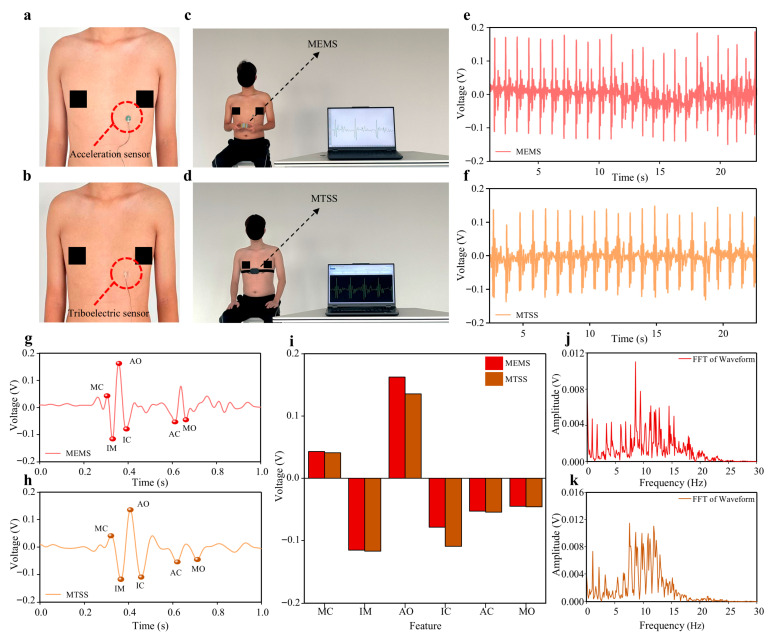
Comparison of SCG signals acquired by the flexible sensor and a MEMS sensor. (**a**,**b**) The acceleration sensor and triboelectric sensor were both fixed to the lower-middle region of the sternum. (**c**,**d**) The same individual (a 21-year-old male subject) wore the MEMS device and the MTSS, respectively, for SCG monitoring. (**e**,**f**) SCG waveforms acquired using the MEMS device and the MTSS. (**g**,**h**) Averaged cycle waveforms obtained from the raw SCG signals after data processing. (**i**) Comparison of the ability of the MEMS device and the MTSS to capture SCG feature points (MC, IM, AO, IC, AC, and MO). (**j**,**k**) Frequency spectra of the SCG signals acquired by the MEMS device and the MTSS, respectively.

Accelerometers are currently the mainstream sensors for measuring seismocardiographic (SCG) signals and are capable of accurately capturing subtle cardiac vibrations with high precision [29,30]. To further evaluate the ability of the flexible pressure sensor to acquire standard SCG waveforms while maintaining conformal flexibility, a microelectromechanical system (MEMS) accelerometer was selected as the reference device for comparative experiments. Figure 3a,b shows that both the acceleration sensor and the triboelectric sensor were fixed to the lower-middle region of the sternum. Figure 3c,d show the subject undergoing SCG monitoring using the MEMS device and the MTSS, respectively, while the raw data acquired by the two systems are presented in Figure 3e,f. The data acquisition process is shown in Supplementary Movies S1 and S2.

In the analysis of SCG signals, commonly identified feature points include Mid-Cycle (MC), Isovolumetric Moment (IM), Aortic Valve Opening (AO), Isovolumetric Contraction (IC), Aortic Valve Closure (AC), and Mitral Valve Opening (MO). These feature points correspond to key mechanical events during cardiac contraction and relaxation and help extract important physiological information from SCG signals [31,32,33]. The raw data were therefore processed to obtain averaged cycle waveforms (Figure S9), and the detection results of these feature points for the MEMS sensor and the flexible pressure sensor are shown in Figure 3g and Figure 3h, respectively. The maximum cross-correlation coefficient between the two averaged cycle waveforms was approximately 0.98, indicating strong waveform agreement and good signal fidelity for the SCG signals acquired by the MTSS. Furthermore, comparison of the extracted feature points, as shown in Figure 3i, revealed that the signal amplitudes at the MC, IM, AC, and MO points were similar between the two sensors. However, at the AO point, the voltage recorded by the MEMS sensor was clearly higher than that recorded by the flexible pressure sensor, whereas at the IC point, the flexible pressure sensor exhibited a more prominent response than the MEMS sensor.

Figure 3j,k presents the frequency spectrum analysis results of the two sensors. It can be seen that the main frequency ranges of the MEMS sensor and the flexible pressure sensor were very similar, indicating comparable frequency-response performance. Taken together, these results demonstrate that the flexible pressure sensor can effectively capture SCG signals and extract relevant physiological information. As a flexible device, the flexible pressure sensor offers clear advantages over rigid MEMS sensors in terms of comfort and long-term stability, making it particularly suitable for home-based monitoring scenarios.

### 3.4. Investigation of Inter-Individual Differences in SCG Signals

**Figure 4:**
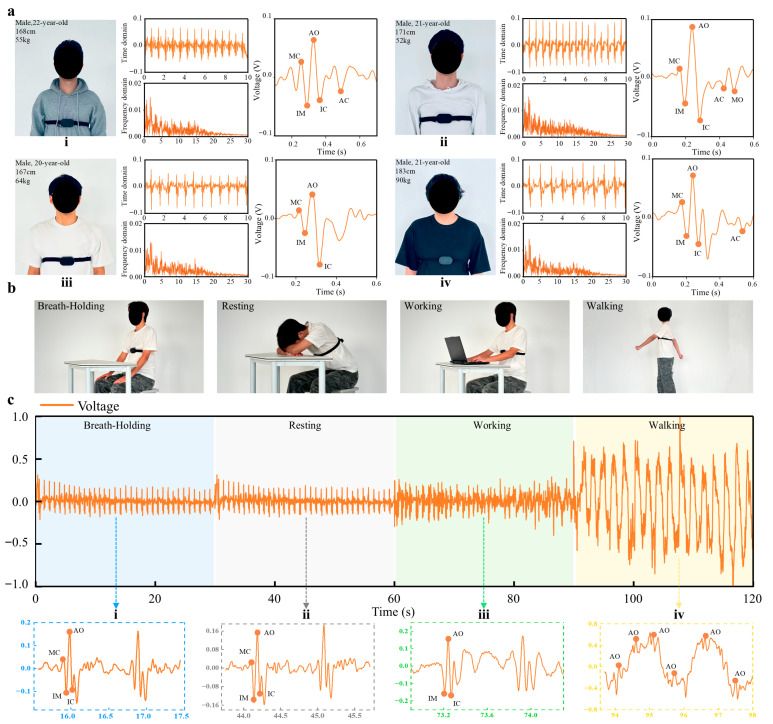
Investigation of differences in SCG signals across individuals and under different daily activity states. (**a**) SCG waveforms from different individuals: (**i**) height 168 cm, weight 55 kg; (**ii**) height 171 cm, weight 52 kg; (**iii**) height 167 cm, weight 64 kg; and (**iv**) height 183 cm, weight 90 kg. (**b**) Schematic illustrations of different activity states. (**c**) SCG waveforms acquired by the MTSS under different activity states: (**i**) breath-holding, (**ii**) resting, (**iii**) working, (**iv**) walking.

Because of differences in cardiac structure and function, as well as individual physical characteristics such as age and body shape, SCG signals from different subjects generally exhibit pronounced inter-individual variations in waveform morphology, amplitude, and spectral distribution [34]. Therefore, four healthy individuals were recruited for the experiment. The subjects differed in age, height, and body weight to ensure a comprehensive evaluation of sensor performance. Each subject underwent 1 min of SCG monitoring under the same experimental conditions. The acquired signals were processed, and the results are shown in Figure 4a. A demonstration of part of the data acquisition process is provided in Supplementary Movies S3 and S4.

A comparative analysis of the SCG signals from the four subjects revealed clear inter-individual differences. Specifically, as shown in Figure 4a(ii), the SCG signal of one subject was able to capture feature points such as AC and MO, whereas in Figure 4a(iii), some feature points were missing from the SCG signal of another subject. This difference may arise from inter-individual variations in chest wall mechanical properties and cardiac structure and function, which can cause certain features to appear weaker or to be less stably distinguishable in some subjects. Nevertheless, the SCG signals from all four subjects showed that feature points such as MC, IM, AO, and IC could be clearly identified. These results indicate that the flexible pressure sensor can be used to capture key features of SCG signals, thereby providing reliable technical support for personalized health monitoring.

### 3.5. Differences in Seismocardiographic Signals Under Different Activity States

Previous studies have shown that different physical activity states can alter cardiac physiological processes, thereby leading to corresponding changes in SCG signal characteristics [35,36]. At the same time, SCG is relatively sensitive to motion artifacts and is therefore susceptible to interference from body movement [37,38]. To verify the effectiveness of the MTSS in real-life scenarios, we selected a healthy 21-year-old subject who wore the MTSS device for SCG acquisition under different activity states. Before testing, the subject rested for 10 min to ensure that the heart rate returned to a calm state, and then sequentially performed four conditions—breath-holding, resting, office work, and walking (Figure 4b)—with each condition lasting 30 s and a 10 min interval between conditions to ensure data reliability.

Under the breath-holding and resting conditions, because body movement was minimal and heart rate fluctuations were small, the variations in the SCG signals were relatively stable and less affected by motion interference, allowing clear capture of feature points such as MC, IM, AO, and IC. Under these two conditions, the SCG signals showed strong periodicity, and under the breath-holding condition, the reduced heart rate made the periodic variation in the SCG signal more pronounced. The SCG cycle under the breath-holding condition was approximately 1 s [Figure 4c(i)], whereas under the resting condition it was approximately 0.8 s [Figure 4c(ii)], and the peak-to-peak amplitude in the breath-holding condition was significantly greater than that in the resting condition. This phenomenon indicates that the MTSS can sensitively capture changes in SCG signals caused by a reduction in heart rate.

During office work, the subject maintained a seated posture with little activity, and the cardiac and respiratory rhythms were relatively stable. However, subtle postural changes, such as movements of the neck, shoulders, and hands, may introduce low-frequency artifacts (0.5–2 Hz), which could affect signal quality. Nevertheless, although the signal quality was slightly inferior to that under the breath-holding and resting conditions, the MTSS was still able to extract feature points from the SCG signal, such as AO, IM, and IC [Figure 4c(iii)], for further physiological state analysis.

Under the walking condition, body movement introduced relatively strong mechanical vibrations, particularly those associated with vertical oscillation, which affected the SCG signal. However, the increased cardiac pumping activity during walking enhanced the vibratory components in the SCG signal. The superposition of step-related vibrations and the periodic fluctuations of the SCG signal made the waveform more complex. As shown in the figure, in the SCG waveform recorded during walking, the large-amplitude fluctuations mainly originated from walking-induced mechanical vibrations, but the AO peak in the SCG signal could still be clearly identified within these fluctuations [Figure 4c(iv)]. The current system is primarily suitable for signal monitoring under resting or low-motion conditions. By improving the conformal contact between the device and the skin and integrating signal filtering, motion-induced interference can be mitigated to some extent, thereby enabling the extraction of relevant features during low-intensity daily activities, which is of considerable significance for physiological signal monitoring in everyday settings.

### 3.6. Machine Learning Models for SCG and Blood Pressure

**Figure 5:**
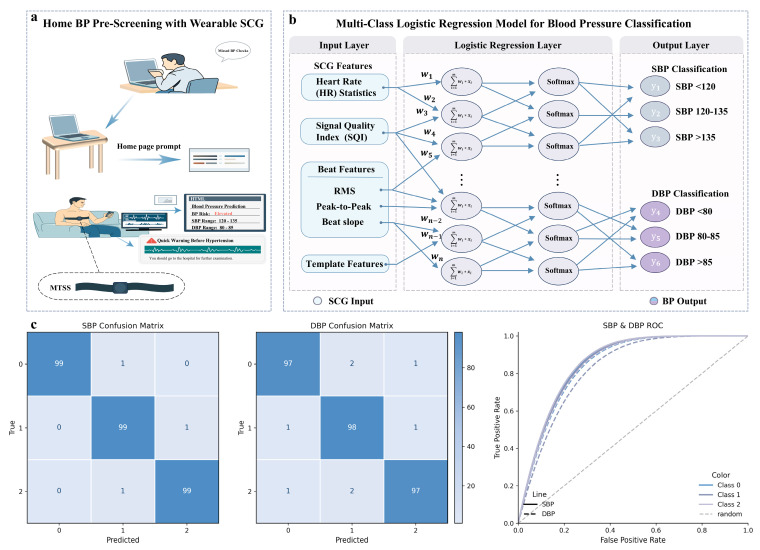
Machine learning models for SCG and blood pressure. (**a**) The MTSS supports SCG monitoring in home-based settings and enables graded early warning for blood pressure abnormalities based on the acquired SCG signals. (**b**) Architecture of the three-class classification models for SBP and DBP. (**c**) Performance evaluation of the systolic (SBP) and diastolic (DBP) blood pressure classification models. Model performance was assessed using confusion matrices and receiver operating characteristic (ROC) curves. Class labels are defined as follows: for SBP, 0: <120 mmHg, 1: 120–135 mmHg, 2: >135 mmHg; for DBP, 0: <80 mmHg, 1: 80–85 mmHg, 2: >85 mmHg.

The preceding experiments in this study have verified the effectiveness of the MTSS in acquiring seismocardiographic (SCG) signals and extracting key feature points. To further explore the practical application potential of SCG features, we investigated the relationship between SCG signals and blood pressure. Essentially, the relationship between SCG and blood pressure can be regarded as the manifestation of “mechanical pumping events” within the cardiac cycle, which generate blood flow and pressure waves that are reflected in the vasculature as blood pressure/pulse waveforms. Therefore, SCG and blood pressure represent adjacent links in the same causal chain [39,40], rather than a simple linear relationship. To establish a predictive model between SCG and blood pressure, this study used SCG signals acquired by the MTSS and synchronously recorded blood pressure data with an upper-arm cuff-based electronic sphygmomanometer (Figure S10), thereby constructing a supervised learning model. As shown in the application scenario shown in Figure 5a, when the model output indicates an abnormal blood pressure warning, the subject would be advised to visit a hospital for further examination, thereby enabling continuous preliminary monitoring of blood pressure in home settings. This application scenario fully demonstrates the value and potential of SCG features. Currently, the MTSS informs users of their blood pressure status through a computer interface. Data processing and display are conducted on the host computer (Figure S11). Future versions may integrate mobile application support to enhance user convenience and experience.

We defined the time point of each cuff-based blood pressure measurement as t_0_, and the preceding 45 s of SCG data were extracted as one sample window. The systolic blood pressure (SBP) and diastolic blood pressure (DBP) measured at t_0_ were used as labels and discretized into a three-class classification task. Separate three-class models were then trained for SBP and DBP. As shown in Figure S12, considering that wearing pressure and contact conditions may cause differences in signal amplitude, the data were normalized and standardized during subsequent processing. The morphological features of SCG signals depend on stable alignment and segmentation of heartbeats. To ensure accurate heartbeat alignment, a three-step procedure was adopted in the heartbeat localization stage. First, the SCG signal was enhanced using a narrow-band band-pass filter to emphasize vibration components related to cardiac activity while simultaneously suppressing low-frequency drift and high-frequency noise. Second, a sliding root mean square (RMS) envelope was calculated to transform the originally morphologically complex, multi-peak vibratory waveform into a smooth energy profile, thereby improving the detectability of “one dominant peak per beat.” Finally, peak detection was performed on the envelope signal, providing a reliable temporal reference for the subsequent calculation of the interval sequence between two adjacent heartbeats (RR intervals) and for single-beat segmentation, thus yielding a set of aligned heartbeats with uniform length.

To reduce training noise caused by motion artifacts and segmentation errors, among other factors, we constructed a signal quality index (SQI), as detailed in Table S1. When the SQI value was lower than 0.7, the corresponding window was labeled as low quality and excluded. For the valid windows retained after SQI-based screening, multiple feature vectors were extracted, including rhythm-related features (e.g., heart rate), heartbeat intensity and dynamic features (e.g., RMS intensity, peak-to-peak amplitude, and maximum upstroke slope of each beat), as well as template features. The latter were obtained by aligning and averaging multiple beats within one window to generate a heartbeat waveform template, from which the amplitudes at 0.1 s, 0.2 s, 0.3 s, and 0.4 s were taken as features. Model training was performed using multinomial logistic regression, and the probability of each class was output through the Softmax function. The final output classes are shown in Figure 5b.

To evaluate model performance, the dataset was randomly divided into a training set and a test set, with the test set accounting for 30% of the data. The models were evaluated using confusion matrices and receiver operating characteristic (ROC) curves. As shown in Figure 5c, the SBP model achieved a classification accuracy of 99%, whereas the DBP model achieved a classification accuracy of 97%. Ultimately, by acquiring SCG signals through the MTSS device and predicting the ranges of blood pressure values (SBP and DBP), the application potential of SCG signals in real-world scenarios was validated.

## 4. Conclusions

In this study, we introduced a flexible pressure sensor for seismocardiographic signal monitoring. Based on the coupled mechanisms of contact electrification and electrostatic induction, the sensor can convert subtle vibrations on the sternum surface induced by cardiac mechanical activity into measurable electrical signals, which are subsequently acquired and analyzed by the MTSS. Performance evaluation demonstrated that the sensor exhibits high responsiveness in the small-force range, with a sensitivity of up to 3.76 V/N over 0–0.2 N. It also showed good low-frequency response characteristics over the 0.5–25 Hz band, covering the main frequency components of SCG signals. In addition, the sensor maintained a stable output after approximately 5300 cycles, indicating good mechanical durability. Based on these characteristics, the MTSS can be integrated into a wearable strap structure, such as a Velcro strap, and fixed to the lower-middle segment of the sternum to achieve continuous acquisition of SCG signals from different individuals under different daily conditions. Furthermore, typical feature points such as AO, IM, and IC could be stably identified from the SCG waveforms obtained by the MTSS, enabling physiological event annotation and state analysis. On this basis, we constructed three-class machine learning models for systolic blood pressure (SBP) and diastolic blood pressure (DBP), which mapped synchronously acquired SCG signals to their corresponding blood pressure ranges. The resulting accuracies reached 99% and 97%, respectively, providing a feasible solution for blood pressure risk warning and health management in home-based settings. Overall, the MTSS proposed in this study provides a new approach and experimental evidence for the application of flexible pressure sensors in the continuous monitoring of vital signs in daily life.

## Data Availability

The data that support the findings of this study are available from the corresponding author upon reasonable request.

## Supplementary Materials

The following supporting information can be downloaded at: https://www.mdpi.com/article/10.3390/bios16050260/s1, Figure S1. (a) The sensor is installed on the side of the Velcro near the heart. (b) The conductive fibers are connected to the ground of the circuit board with the human body as the reference point. Figure S2. Third-order low-pass Butterworth filter circuit (a) and its frequency response (b). Figure S3. (a) A 200 μm thick PET film was used as the base. (b) The PET double-sided adhesive is cut into circular rings to serve as the spacer layer. (c) PET square pad (Area: 1∙1 [mm]^2^). Figure S4. SEM images of PTFE powder at different magnifications. (a) SEM image of PTFE powder with a scale bar of 5 μm, showing the general morphology and surface structure of the particles. (b) SEM image of PTFE powder with a scale bar of 2 μm, providing a closer view of the particle surface and highlighting finer details of the material’s texture. Figure S5. Effect of PTFE powder amount on the sensor output voltage under different applied pressures. The voltage response of the sensor with 20 mg, 40 mg, and 60 mg PTFE powder amounts is shown at various applied pressures (0.13 N, 0.27 N, 0.5 N, and 1 N). The data indicate that the voltage increases with pressure, with the highest output observed at 40 mg of PTFE powder. Figure S6. Relationship between load pressure and maximum short-circuit current (Isc). As the applied load pressure increases, the maximum short-circuit current (Isc) increases linearly, indicating that the enhanced charge generation is due to the tighter contact between the sensor surface and the electrodes. Figure S7. Effect of external load resistance on the output voltage and power of the sensor. Figure S8. Measurement of response and recovery times. Response time: The time taken for the sensor’s current to reach its maximum value after the application of pressure. Recovery time: The time required for the sensor’s maximum reverse current to return to its baseline value after the removal of pressure. Figure S9. Average Period Waveform Processing Flowchart. Figure S10. Participants’ systolic blood pressure (SBP) and diastolic blood pressure (DBP) were synchronously acquired using a cuff-based blood pressure monitor, together with seismocardiogram (SCG) signals recorded via the MTSS system. Figure S11. The waveform of the SCG signal from the host computer and the classification results of SBP/DBP into three categories. Figure S12. Flowchart of the three-category modeling process for SBP/DBP. Table S1. We employed a signal quality index (SQI) to assess whether the acquired SCG recordings were suitable for model training. Movie S1. Monitoring SCG signals using the MTSS. Movie S2. Monitoring SCG signals using the MEMS. Movies S3 and S4. Individual differences in SCG signals.
