# Supplementary material for: A Flexible Triboelectric-Based Sensor for Seismocardiography Monitoring
Source: Biosensors (Basel). 2026 May 1;16(5):260. doi: 10.3390/bios16050260 (PMC13204470; doi:10.3390/bios16050260)
Supplement: Supplementary file 1 [file biosensors-16-00260-s001.zip › biosensors-4223251-supplementary.pdf]

A Flexible Triboelectric-Based Sensor for Seismocardiography Monitoring

Changke Wang, Yingjie He, Haojie Peng, Haijun Luo * and Xue Wang *

College of Physics and Optoelectronic Engineering, Chongqing Normal University, Chongqing 401331, China

*Authors to whom correspondence should be addressed. Electronic mail:

Email: 20132098@cqnu.edu.cn (H.L.); wangxue@cqnu.edu.cn (X.W.)

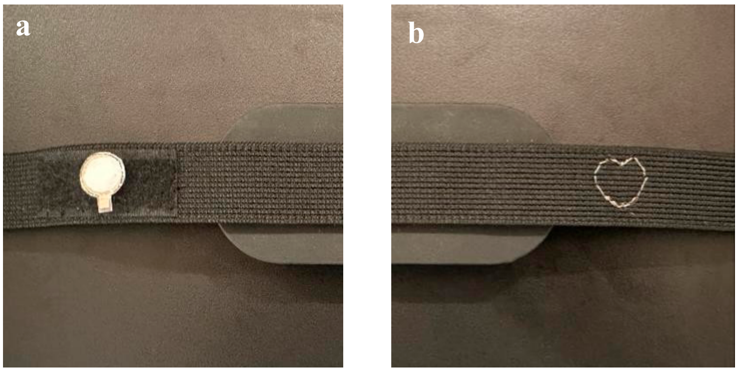

Fig. S1. (a) The sensor is installed on the side of the Velcro near the heart. (b) The conductive fibers are connected to the ground of the circuit board with the human body as the reference point.

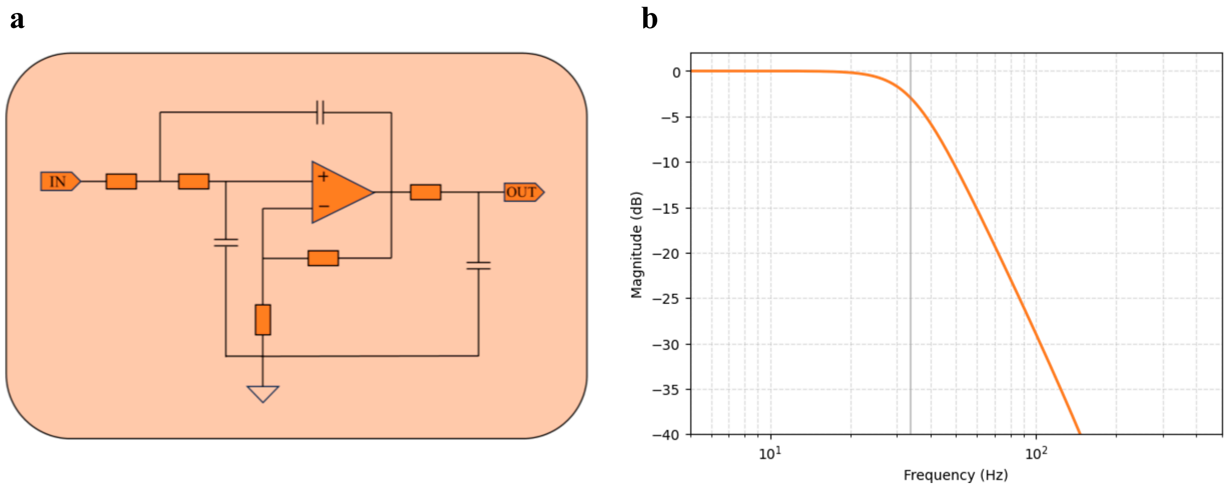

Fig. S2. Third-order low-pass Butterworth filter circuit (a) and its frequency response (b).

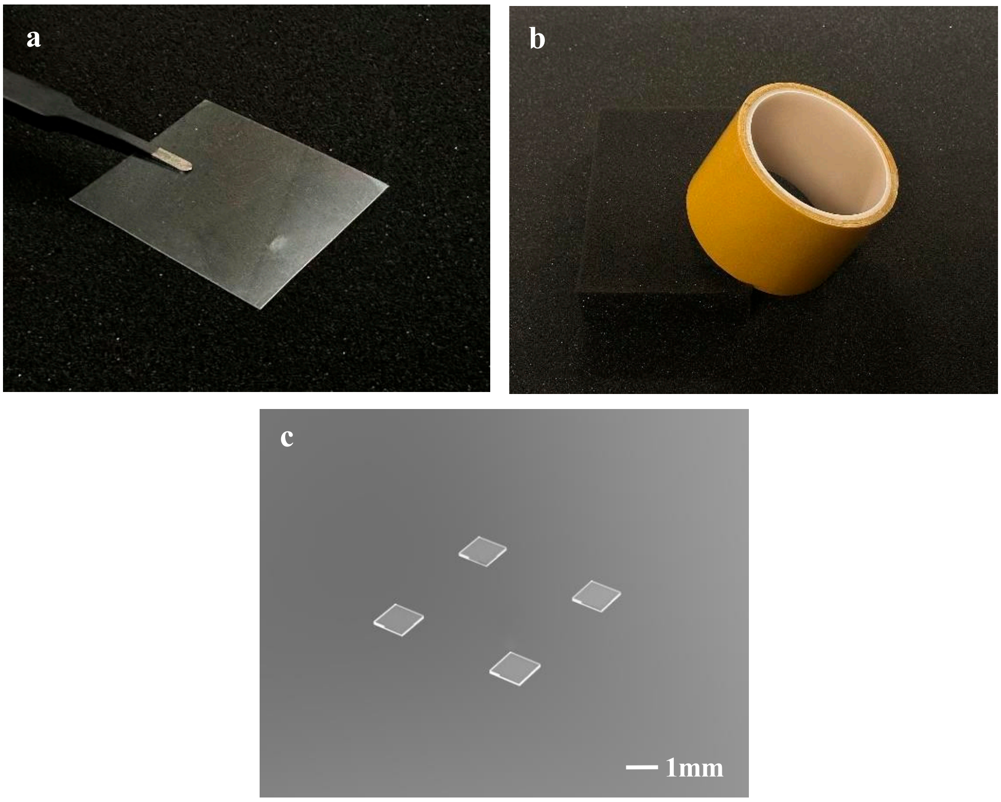

Fig. S3. (a) 200 μm thick PET film was used as the base. (b) The PET double-sided adhesive is cut into circular rings to serve as the spacer layer. (c) PET square pad (Area: $1 \cdot 1mm^2$).

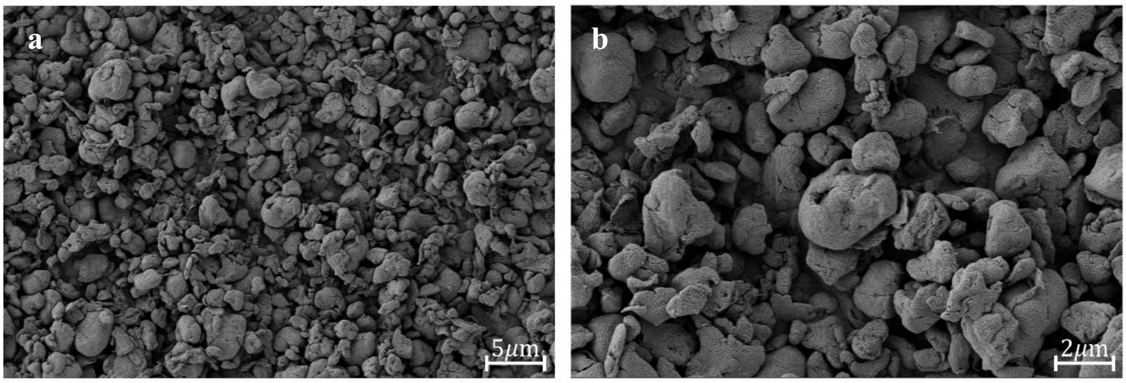

Fig. S4. SEM images of PTFE powder at different magnifications. (a) SEM image of PTFE powder with a scale bar of 5 μm, showing the general morphology and surface structure of the particles. (b) SEM image of PTFE powder with a scale bar of 2 μm, providing a closer view of the particle surface and highlighting finer details of the material's texture.

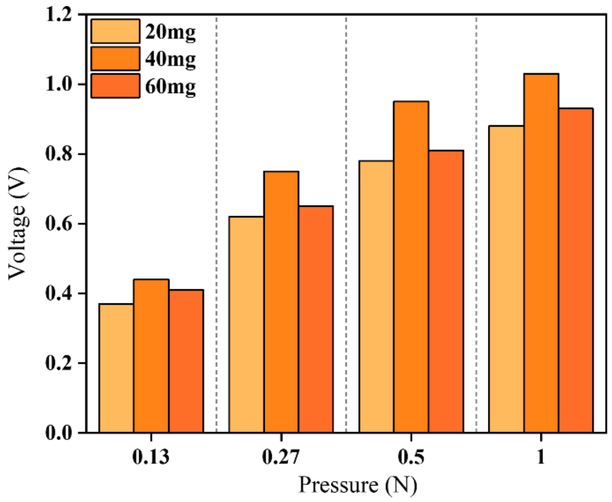

Fig. S5. Effect of PTFE powder amount on the sensor output voltage under different applied pressures. The voltage response of the sensor with 20mg, 40mg, and 60mg PTFE powder amounts is shown at various applied pressures (0.13 N, 0.27 N, 0.5 N, and 1 N). The data indicate that the voltage increases with pressure, with the highest output observed at 40mg of PTFE powder.

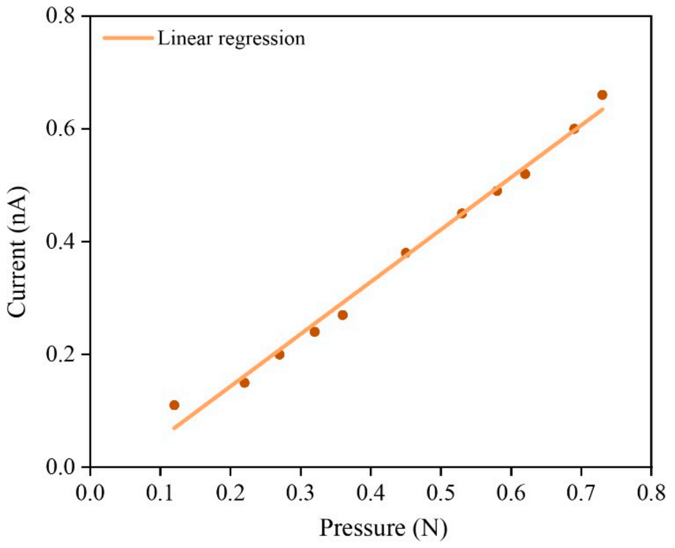

Fig. S6. Relationship between load pressure and maximum short-circuit current (Isc). As the applied load pressure increases, the maximum short-circuit current (Isc) increases linearly, indicating that the enhanced charge generation is due to the tighter contact between the sensor surface and the electrodes.

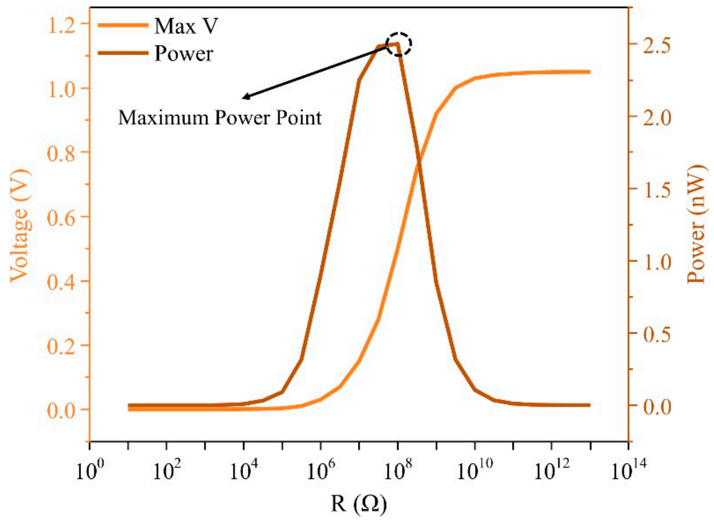

Fig. S7. Effect of external load resistance on the output voltage and power of the sensor.

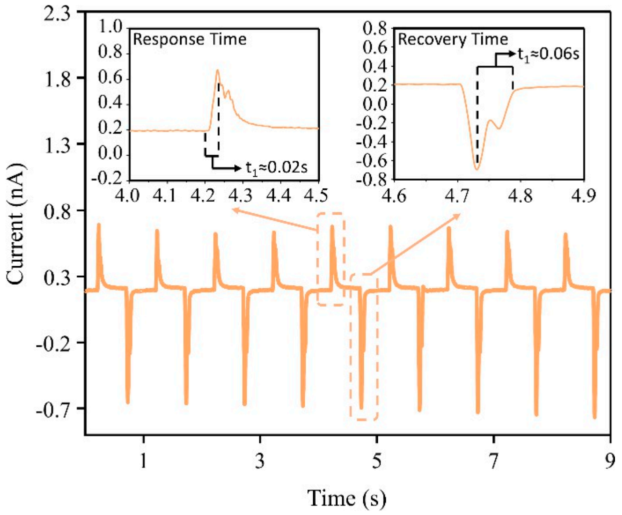

Fig. S8. Measurement of response and recovery times. Response time: The time taken for the sensor's current to reach its maximum value after the application of pressure. Recovery time: The time required for the sensor's maximum reverse current to return to its baseline value after the removal of pressure.

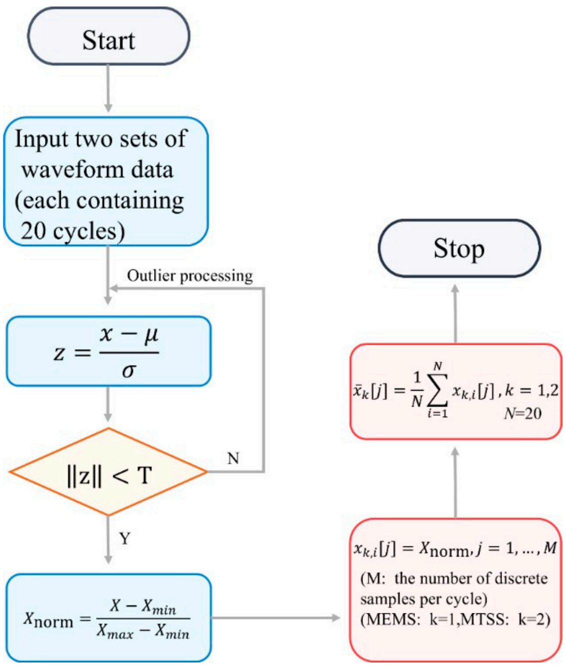

Fig. S9. Average Period Waveform Processing Flowchart.

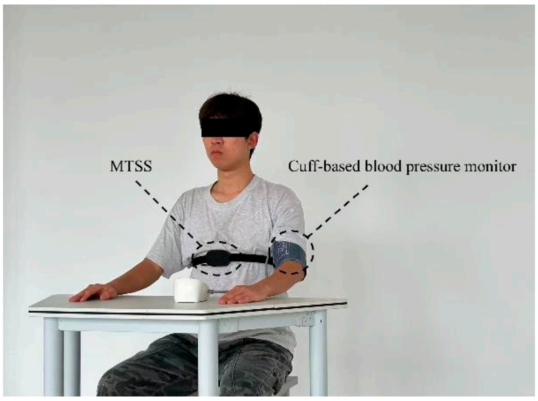

Fig. S10. Participants' systolic blood pressure (SBP) and diastolic blood pressure (DBP) were synchronously acquired using an cuff-based blood pressure monitor, together with seismocardiogram (SCG) signals recorded via the MTSS system.

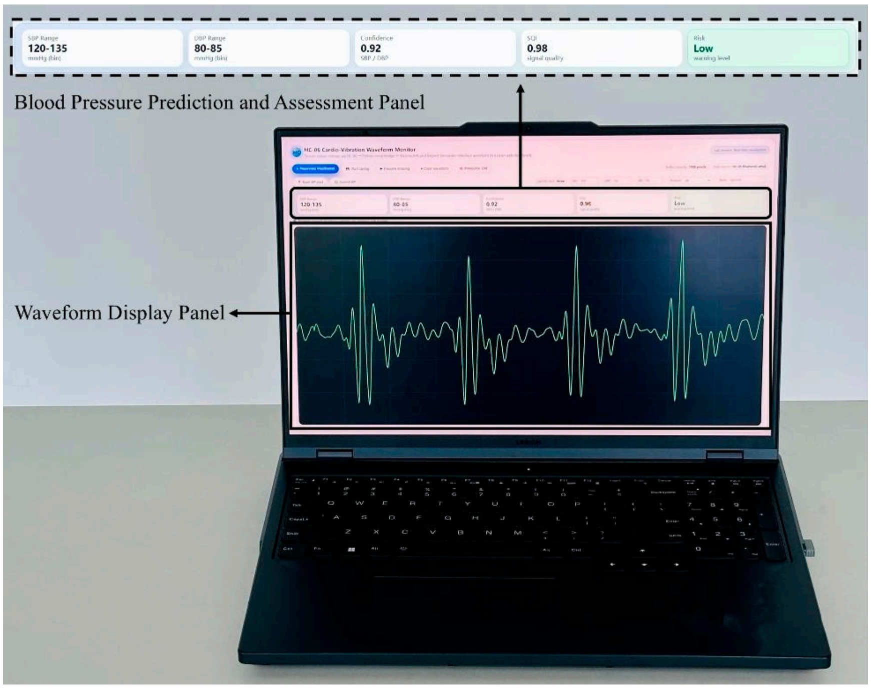

Fig. S11. The waveform of the SCG signal from the host computer and the classification results of SBP/DBP into three categories.

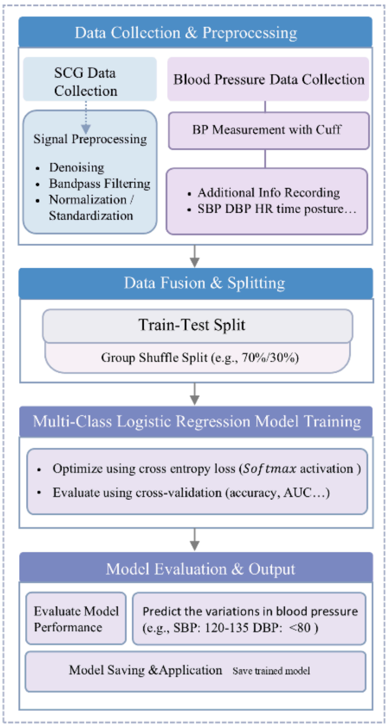

Fig. S12. Flowchart of the three-category modeling process for SBP/DBP.

Table. S1. We employed a signal quality index (SQI) to assess whether the acquired SCG recordings were suitable for model training.

Symbol	Description	Formula	Notes
SQI	Signal Quality Index	$SQI = w_{corr} \cdot SQI_{corr} + w_{rr} \cdot SQI_{rr}$	Composite index of overall signal fidelity
w_{corr}, w_{rr}	Weighting coefficients	$w_{corr} = 0.7, w_{rr} = 0.3$	Weights for the morphological and RR-interval stability components
SQI_{corr}	Morphological Consistency Score	$SQI_{corr} = median(\{c_k\}_{k=1}^{K})$	Median of the correlation coefficients between individual beats and the template
c_k	Normalized correlation	$c_k = \frac{\sum_{n=1}^{N} \tilde{b}_k[n] \cdot \tilde{t}[n]}{\sqrt{\sum_{n=1}^{N} \tilde{b}_k[n]^2} \cdot \sqrt{\sum_{n=1}^{N} \tilde{t}[n]^2}}$	$\tilde{b}_k[n]$ and $\tilde{t}[n]$ are the zero-mean heartbeat segment and template, respectively
$t[n]$	Average beat template	$t[n] = \frac{1}{K}\sum_{k=1}^{K} b_k[n]$	Constructed by averaging K heartbeat segments $b_k[n]$ within the analysis window
SQI_{rr}	RR Interval Stability Score	$SQI_{rr} = exp(-\alpha \cdot CV_{RR}), \alpha = 8.0$	α is a scaling parameter (set to 8.0 in this study)
CV_{RR}	Coefficient of Variation of RR intervals	$CV_{RR} = \frac{\sigma_{RR}}{\mu_{RR}}$	Ratio of the standard deviation (σ_{RR}) to the mean (μ_{RR}) of RR intervals
RR_i	the i-th RR interval	$RR_i = \frac{p_{i+1} - p_i}{f_s}, i = 1,2,\ldots,M$	Derived from envelope peak positions p_i and the sampling frequency f_s

K: number of heartbeat segments in the analysis window.

N: number of samples in each heartbeat segment $b_k[n]$

M: total number of RR intervals.
